# Intrathoracic eosinophilic sclerosing fibroplasia with intralesional bacteria in a cat

**DOI:** 10.1177/20551169231199447

**Published:** 2023-11-01

**Authors:** Antoine A Duclos, Alan Wolfe, Carmel T Mooney

**Affiliations:** 1 Small Animal Clinical Studies, School of Veterinary Medicine, University College Dublin, Dublin, Ireland; 2 Pathobiology, School of Veterinary Medicine, University College Dublin, Dublin, Ireland

**Keywords:** Eosinophilic sclerosing fibroplasia, mediastinal mass, microbiology, eosinophilic inflammation

## Abstract

**Case summary:**

A 9-year-old neutered female domestic shorthair cat was presented for investigation of a cranial mediastinal mass. Moderate peripheral eosinophilia and mild-to-moderate polyclonal gammopathy were identified. A thoracoabdominal CT scan documented a cranial mediastinal mass encircling the trachea. Ultrasound-guided fine-needle aspiration and core-needle biopsy were performed, but cytology and histopathology were inconclusive. Surgical debulking was performed. Further histological samples identified severe pyogranulomatous and eosinophilic fibrosing mediastinitis, consistent with feline eosinophilic sclerosing fibroplasia. Gram staining and fluorescence in situ hybridisation (FISH) identified numerous Gram-positive coccoid bacteria. Eosinophilia and hyperglobulinaemia resolved after surgery and combined antimicrobial and immunosuppressive therapy. The cat died 3 months later after developing acute haemorrhagic diarrhoea and dyspnoea.

**Relevance and novel information:**

Eosinophilic sclerosing fibroplasia is reportedly mainly confined to the gastrointestinal tract in cats. Less commonly, extragastrointestinal cases have been described. Lesions in the mediastinal or sternal lymph nodes have been reported, all in association with evident gastrointestinal involvement. The presence of pleural effusion was variable in these cases. To the authors’ knowledge, this is the first report of eosinophilic sclerosing fibroplasia presenting due to lower respiratory signs in a cat. Intralesional bacteria were identified using Gram staining and FISH examination. The presence of intralesional bacteria in the normally sterile mediastinal tissue may support the involvement of penetrating injuries in the pathogenesis of the disease. Eosinophilic sclerosing fibroplasia should be suspected in any cat with abdominal and/or thoracic masses, particularly if associated with peripheral eosinophilia and polyclonal gammopathy.

## Introduction

Feline eosinophilic sclerosing fibroplasia (FESF) is an emerging clinical entity,^1^ with 53 cases reported since its first official characterisation in 2009.^1–14^ Most cases manifest as masses confined to the gastrointestinal tract (GIT), with possible mesenteric lymph node involvement.^2,3,5^ Rarely, the liver, pancreas and sternal or mediastinal lymph nodes are concurrently affected.^1,2,5,6,11^ Key histopathological features include trabeculae of dense collagen separated by large fibroblasts, as well as marked eosinophilic inflammation.^2^ The pathogenesis is unknown, but antigenic stimulation triggering abnormal inflammation in genetically predisposed cats is suspected.^2^ Infectious agents (mainly bacteria) are commonly, but not always, identified in these lesions.^1–3,5,6,10,14,15^ Infection may be the inciting cause of dysregulated inflammation. However, due to the GIT location, mucosal alteration in a non-sterile environment may alternatively promote secondary infection. Treatment includes surgical resection, and immunosuppressive and antimicrobial therapies with variable prognoses.^1,2,5^

To the authors’ knowledge, this is the first case of FESF presenting due to lower respiratory signs caused by a mediastinal mass. Intralesional bacteria were documented in this case, supporting that primary infection could be the driver for a dysregulated immune-mediated response in the pathogenesis of FESF.

## Case description

A 9-year-old neutered female domestic shorthair cat was referred for investigation of a cranial mediastinal mass identified on thoracic radiographs after a 5-week history of cough and a 3-day history of acute-onset dyspnoea characterised by expiratory effort, tachypnoea and open-mouth breathing. Rapid immunoassays for the detection of feline immunodeficiency virus (FIV) antibody and feline leukaemia virus (FeLV) antigen were negative. Non-steroidal anti-inflammatory and antimicrobial therapies were administered, resulting in the resolution of clinical signs. Repeat thoracic radiography demonstrated persistence of the mass 1 week later, prompting referral.

On presentation, the physical examination was unremarkable. Full haematology and biochemistry (Veterinary Diagnostic Laboratory, UCD) identified eosinophilia (eosinophils 2.68 × 10^9^/l, reference interval [RI] 0–1.5 × 10^9^) and hyperglobulinaemia (globulin 60.6 g/l, RI 24–40 ) confirmed as polyclonal by serum protein electrophoresis. The remaining parameters were within their respective RIs. Toxoplasma serology was not consistent with active infection (IgG >800, IgM <20, indirect fluorescence assay).

**Figure 1:**
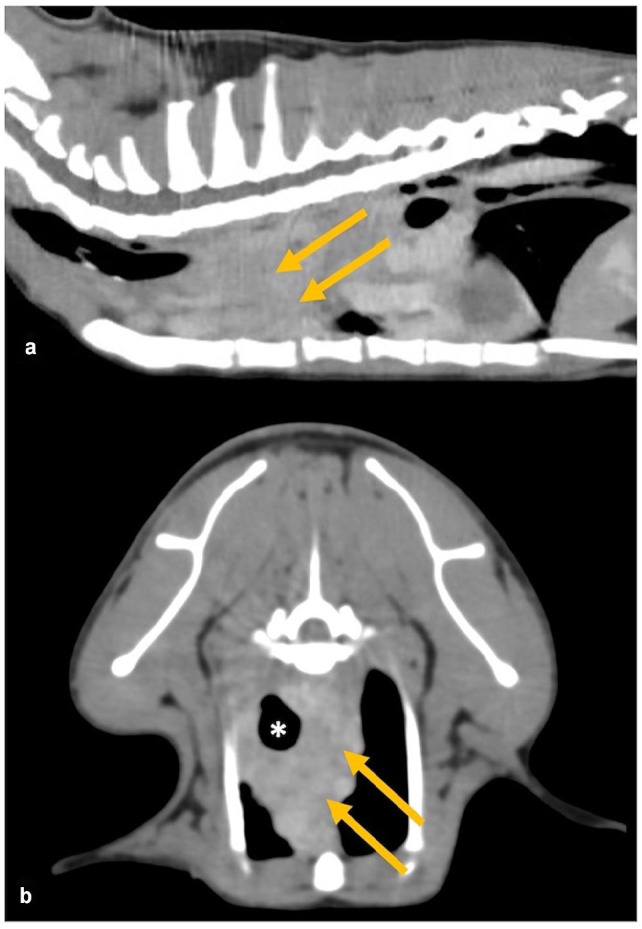
Examples of multiplanar reconstruction in (a) sagittal and (b) transverse images obtained by thoracoabdominal CT after contrast demonstrating the presence of a minimally contrast-enhancing, soft tissue-attenuating mass within the cranial mediastinum (arrows) surrounding and displacing the cranial thoracic trachea (asterisk) to the right

Thoracoabdominal CT identified a minimally contrast-enhancing, soft tissue-attenuating mass within the cranial mediastinum (4.3 × 2.5 × 2.3 cm) surrounding the cranial thoracic trachea (Figure 1). The thoracic lymph nodes and the alimentary tract, including the oesophagus, were unremarkable. Thoracic ultrasonography identified a heterogeneous hyperechoic structure circumferentially surrounding the trachea. Cytology (ultrasound-guided fine-needle aspiration) was acellular and thus inconclusive. Histopathology (ultrasound-guided core biopsy) identified a non-specific inflammatory process with plasma cells, macrophages, lymphocytes and few neutrophils.

Given that an underlying neoplastic process could not be ruled out, further invasive investigation was recommended. The mass was surgically debulked after exploratory sternotomy. As the mass was adherent to the trachea, complete removal was not performed. Impression cytology of the mass identified mixed neutrophilic and eosinophilic inflammation, and extracellular particles resembling cocci in size and shape.

**Figure 2:**
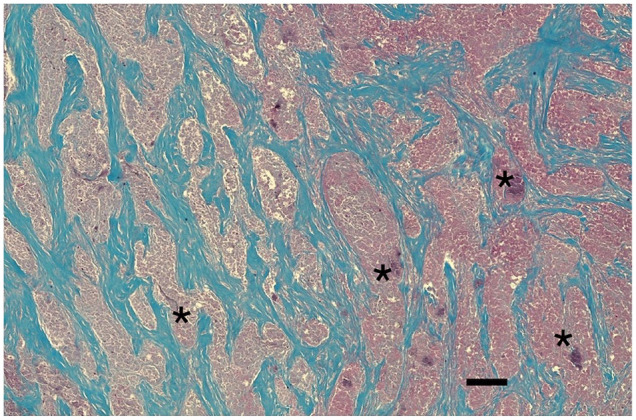
Photomicrograph of an excisional biopsy of a peritracheal mass in the cat, demonstrating characteristic dense streams of collagenous and fibrous tissue (staining blue), multifocally disrupted by numerous eosinophils, lymphocytes, plasma cells, neutrophils, macrophages and multinucleated cells, and associated numerous proliferating blood vessels with necrotic areas (asterisks). Masson’s trichrome stain. Scale bar = 200 µm

Histopathology identified dense streams of collagenous tissue multifocally disrupted by numerous eosinophils, lymphocytes, plasma cells, neutrophils, macro-phages and multinucleated cells, and associated numerous proliferating blood vessels with necrotic areas (Figure 2). These features were consistent with FESF.

**Figure 3:**
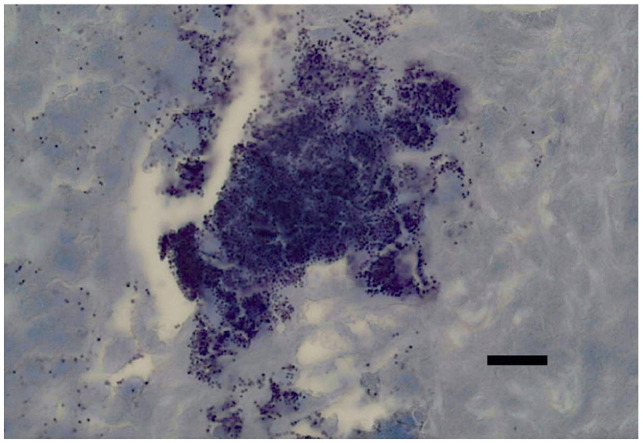
Photomicrograph of an excisional biopsy of the peritracheal mass, demonstrating numerous Gram-positive bacteria (purple colour). Gram stain. Scale bar = 10 µm

**Figure 4:**
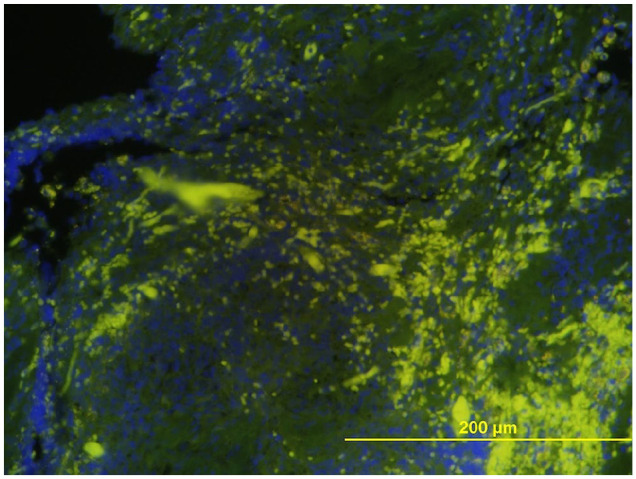
Photomicrograph of the peritracheal mass histopathology demonstrating a large number of invasive bacteria (green fluorescence) through fluorescence in situ hybridisation examination. Animal Health Diagnostic Center, Cornell University

Ziehl–Neelsen stain was negative for acid-fast organisms. Periodic acid Schiff stain did not highlight fungal agents. Toluidine blue stain highlighted only a few scattered mast cells. Gram staining (Figure 3) and FISH examination (Figure 4) using eubacteria probe (EUB338) were simultaneously performed to maximise the sensitivity for bacterial detection, given the cytology results. Numerous Gram-positive coccoid bacteria were identified.

The cat was treated with broad-spectrum antimicrobials (amoxicillin clavulanic acid, 20 mg/kg PO q12h) given the bacterial infection. Immunosuppressive prednisolone therapy (2 mg/kg PO q24h) was administered after the diagnosis of FESF as a benefit of steroid therapy over surgery and antimicrobial therapy alone has been demonstrated.^2^ Three weeks later, the eosinophilia and hyperglobulinaemia had resolved (eosinophils 0.94 × 10^9^/l, globulin 36.5 g/l). Antimicrobial therapy was discontinued 2 weeks later, and a repeat thoracic CT scan 2 months later was recommended.

The cat developed severe haemorrhagic diarrhoea after a further 4 weeks. Thoracic radiographs at the referring veterinarian identified mild pleural effusion and a localised alveolar pattern in the caudo-dorsal lung lobes, without evidence of a cranial mediastinal mass. The cat died shortly after presentation to the referring veterinarian. Further investigations (including post-mortem examination) were not performed.

## Discussion

This case report describes a cat presenting with a cranial mediastinal mass that had histopathological features consistent with FEFS, without obvious evidence of GIT involvement. FEFS presenting with lower respiratory signs has not yet been reported.

Extragastrointestinal cases of FESF have been reported, albeit rarely. Two such cases have been reported with intra-abdominal lesions that were confined to the mesentery or retroperitoneal space, respectively.^9,10^ Another cat had involvement of the pancreas and liver, although with concurrent GIT (pyloric) involvement.^11^ One further cat presented with a nasal mass.^13^ Two additional reports described an intrathoracic location with mediastinal lymphadenopathy either with or without pleural effusion. Evident GIT involvement was concurrently demonstrated in these cases, and the intrathoracic involvement was suspected to have occurred through dissemination via the sternal lymph node.^1,7^ In the case reported herein, an abdominal ultrasound was not performed. Given the initial lack of gastrointestinal signs reported, the unremarkable abdominal palpation and normal abdominal CT scan, it is unlikely that a GIT mass was missed. However, it cannot be ruled out that a microscopic lesion was missed. Before the cat’s death, it developed gastrointestinal signs. A post-mortem examination was not performed. Therefore, it cannot be fully ruled out that a GIT lesion was undetected.

In contrast to previous reports of intrathoracic FESF, the mass did not appear to arise from a thoracic lymph node, based on location upon CT examination and given that histologic features did not suggest that the mass was within a lymph node. However, as the mass was incompletely excised, this possibility cannot be definitively ruled out.

The cat displayed peripheral eosinophilia and hyperglobulinaemia, two of the common clinicopathological features of FESF, documented in 25–58% and 50–64% of reported cases, respectively.^1,2,5^

Intralesional bacteria were identified in this case, similar to other reports.^1–3,9,13^ Rarely, other intralesional infectious agents (nematode and fungi) are also reported.^6,8,14^ The role of infectious agents in the pathogenesis of FESF is unclear. It has been suggested that they represent a primary, inciting trigger for an exaggerated inflammatory response, or alternatively could merely reflect secondary opportunistic infection in a non-sterile environment with abnormal tissue architecture and possible breach in mucosal integrity.^1,2^ The former possibility is supported by a report documenting bacterial-induced subcutaneous and mesenteric masses with histopathologic features resembling FESF.^15^ The latter is supported by the fact that intralesional bacteria are not always identified.^1^ However, cats are often presented for referral after having received antimicrobial therapy. In addition, a combination of culture, special stains (eg, Gram) and FISH examination is not always performed, and bacterial involvement therefore cannot always be fully ruled out.^2,4,5,7,8,10,11^ As illustrated in this case and others, failure to identify infectious agents on standard histopathology does not rule out their presence, and additional testing such as bacterial and fungal cultures, Gram stain or FISH may be required.^1^ In the case presented here, samples for culture were unfortunately not obtained as the initial main suspicion was neoplasia rather than infection.

The primary lesion was located in the cranial mediastinum, normally a sterile environment. This may suggest that bacteria were directly involved in the pathogenesis of the disease rather than representing an opportunistic infection. We can speculate that the bacterial infection may have occurred through a penetrating wound. Although no foreign body was identified in the case, given the peritracheal location of the mass, it is possible that infection occurred secondary to a previous tracheal injury. Prior oesophageal penetration could also be considered, although the CT examination documented a normal oesophagus. Penetrating wounds from migrating foreign bodies have been suspected to be the initial insult facilitating infection in FESF, and it is suspected that in the GIT, pyloric and ileocolic localisation of the lesion is more common because these areas are more likely to be injured by the transit of sharp foreign bodies.^2^ Alternatively, mechanical/traumatic injury rather than infection may also be the initial driver of the dysregulated immune response in the pathogenesis of FESF.

Regardless of the presence of a suspected underlying antigenic trigger, similar to other eosinophilic inflammatory processes, an exaggerated immune response is suspected to be involved in the pathogenesis of the disease.^16^ This is supported by the fact that prolonged survival has been identified in cats with FESF treated with immunosuppressive and antimicrobial therapies as opposed to antimicrobial therapy alone.^2^ The exuberant collagen deposition and fibrosis are likely mediated by the eosinophils and are suspected to involve dysregulation of expression of extracellular matrix protein and transforming growth factor-β_1_.^16^

The reported prognosis in FESF is highly variable, and likely depends on the clinical presentation. Cases presenting with locally aggressive unresectable masses have a poor reported outcome.^1,6,11^ More recent publications suggest a more prolonged survival (>1 year). This is likely because of an earlier diagnosis as a result of increased awareness, as well as improved understanding of the pathogenesis of the condition resulting in more appropriate management.^1,9,12,13^ A multimodal approach is currently recommended with a combination of debulking surgery, when possible, together with antimicrobial and immunosuppressive therapy (prednisolone with or without ciclosporine).^1,9,10,12–14^ Despite adopting this multimodal approach, in the case reported here, death occurred only 2 months after diagnosis. The cause remained undetermined. Antimicrobial therapy had been discontinued 2 weeks before death. Given the incomplete surgical resection and the previously demonstrated presence of bacteria, it is possible that residual infection remained, and that discontinuation of antimicrobial therapy resulted in sepsis. Immuno-suppressants may have also facilitated another opportunistic infection. Therefore, it may be prudent to consider repeat imaging in such cases before discontinuation of antimicrobials. Alternatively, given the reported gastrointestinal signs immediately before death, it is also possible that FESF developed in the GIT despite immunosuppression.

## Conclusions

This is the first case report of intrathoracic FESF presenting with lower respiratory signs due to a cranial mediastinal mass associated with intralesional bacteria. An infectious aetiology should be investigated through specific testing. FESF should be suspected in any cat with abdominal and/or thoracic masses, particularly if associated with peripheral eosinophilia and polyclonal gammopathy. Follow-up imaging should be considered before the discontinuation of antimicrobial therapy. The presence of intralesional bacteria in a normally sterile environment may support the involvement of penetrating injuries in the pathogenesis of the disease.
